# P-Cresylsulfate, the Protein-Bound Uremic Toxin, Increased Endothelial Permeability Partly Mediated by Src-Induced Phosphorylation of VE-Cadherin

**DOI:** 10.3390/toxins12020062

**Published:** 2020-01-21

**Authors:** Shih-Chieh Chen, Shin-Yin Huang, Chia-Chun Wu, Chiung-Fang Hsu

**Affiliations:** 1 Graduate Institute of Medicine, College of Medicine, Kaohsiung Medical University, Kaohsiung 80708, Taiwan; 2 Department of Anatomy, Faculty of Medicine, College of Medicine, Kaohsiung Medical University, Kaohsiung 80708 Taiwan; frea61@yahoo.com.tw (S.-Y.H.); staceyhana@gmail.com (C.-F.H.); 3 Department of Medical Research, Kaohsiung Medical University Hospital, Kaohsiung 80708 Taiwan; 4 Department of Nephrology, Chi Mei Medical Center, Tainan 71004, Taiwan; chiachun4481@gmail.com; 5 Department of Pharmacy, Chia Nan University of Pharmacy and Science, Tainan 71710, Taiwan

**Keywords:** p-cresylsulfate, endothelial permeability, VE-cadherin, chronic kidney disease, Src kinase, endothelial dysfunction

## Abstract

The goal of our study was to investigate the impact of p-cresylsulfate (PCS) on the barrier integrity in human umbilical vein endothelial cell (HUVEC) monolayers and the renal artery of chronic kidney disease (CKD) patients. We measured changes in the transendothelial electrical resistance (TEER) of HUVEC monolayers treated with PCS (0.1–0.2 mM) similar to serum levels of CKD patients. A PCS dose (0.2 mM) significantly decreased TEER over a 48-h period. Both PCS doses (0.1 and 0.2 mM) significantly decreased TEER over a 72-h period. Inter-endothelial gaps were observed in HUVECs following 48 h of PCS treatment by immunofluorescence microscopy. We also determined whether PCS induced the phosphorylation of VE-cadherin at tyrosine 658 (Y658) mediated by the phosphorylation of Src. Phosphorylated VE-cadherin (Y658) and phosphorylated Src levels were significantly higher when the cells were treated with 0.1 and 0.2 mM PCS, respectively, compared to the controls. The endothelial barrier dysfunction in the arterial intima in CKD patients was evaluated by endothelial leakage of immunoglobulin G (IgG). Increased endothelial leakage of IgG was related to the declining kidney function in CKD patients. Increased endothelial permeability induced by uremic toxins, including PCS, suggests that uremic toxins induce endothelial barrier dysfunction in CKD patients and Src-mediated phosphorylation of VE-cadherin is involved in increased endothelial permeability induced by PCS exposure.

## 1. Introduction

Chronic kidney disease (CKD) is a global health problem. As CKD gradually progresses and the body tissues gradually accumulate uremic toxins, hemodialysis treatment is necessary for CKD patients. However, protein-bound retention solutes such as indoxylsulfate (IS) and p-cresylsulfate (PCS) are not efficiently eliminated by traditional hemodialysis [1,2]. Indole and P-cresol (PC) are derived from amino acids (tyrosine, phenylalanine, and tryptophan) by intestinal bacteria [3], and further modified to generate IS and PCS by intestinal sulfotransferases. Accordingly, the serum levels of both IS and PCS are elevated in CKD and end stage kidney disease (ESKD) dialysis patients [4,5,6]. Furthermore, high serum levels of protein-bound retention solutes such as PCS and IS are associated with increased risk and mortality of cardiovascular disease (CVD) such as atherosclerosis in CKD or hemodialysis patients [7,8,9,10,11,12].

Vascular endothelial cells form the inner lining of blood vessels, and endothelial monolayers function as selective barriers between the blood and surrounding tissues. Endothelial adherens junctions (AJs) are mainly formed by vascular endothelial (VE)-cadherin localized at cell-to-cell junctions. VE-cadherin is a single-span transmembrane protein, and assembles into homomeric dimers to bind VE-cadherin dimers of adjacent cells [13,14,15,16,17]. Tyrosine residues of the cytoplasmic terminus of VE-cadherin can be targets available for tyrosine kinases [13]. Phosphorylation of VE-cadherin followed by disruption of endothelial junctions and the underlying mechanisms of these processes have been the objectives of numerous studies [14,16,18,19,20,21]. Indeed, tyrosine phosphorylation of VE-cadherin and internalization of tyrosine phosphorylated VE-cadherin are also associated with barrier dysfunctions [14,16,18,19].

Src belongs to the Src family of non-receptor protein tyrosine kinases with distinct Src homology (SH) domains [22,23]. In endothelial cells, Src is associated with VE-cadherin in molecular complexes at the AJs [24]. Previous studies indicate that tyrosine phosphorylation of VE-cadherin depends on Src activation under hormone stimulation conditions [24]. Recent studies have also reported that Src is involved in barrier disruption in endothelial cells challenged by inflammatory mediators and growth factors [14,16,22,23,25].

An in situ PCS exposure model of intradermal injection demonstrated increased vascular permeability in skin microvessels in response to PCS stimulation [26]. To investigate the cellular and molecular mechanisms associated with vascular barrier changes induced by PCS exposure, human umbilical vein endothelial cells (HUVECs) were treated with PCS to characterize possible endothelial permeability increases associated with alterations in endothelial junctions and related signaling pathways. Additionally, to further confirm the disrupted endothelial barrier in the arterial intima related to declining kidney function, the endothelial IgG permeability of the renal artery in CKD patients was evaluated. In this study, we demonstrated that non-cytotoxic doses of PCS induced a significant increase in endothelial permeability with concomitant disassembly of intercellular junctions. In addition, disruptions of intercellular junctions were associated with increased levels of phosphorylated VE-cadherin and phosphorylated Src. We also detected increased endothelial leakage of IgG in the arterial intima related to the declining kidney function in CKD patients.

## 2. Results

**Figure 1:**
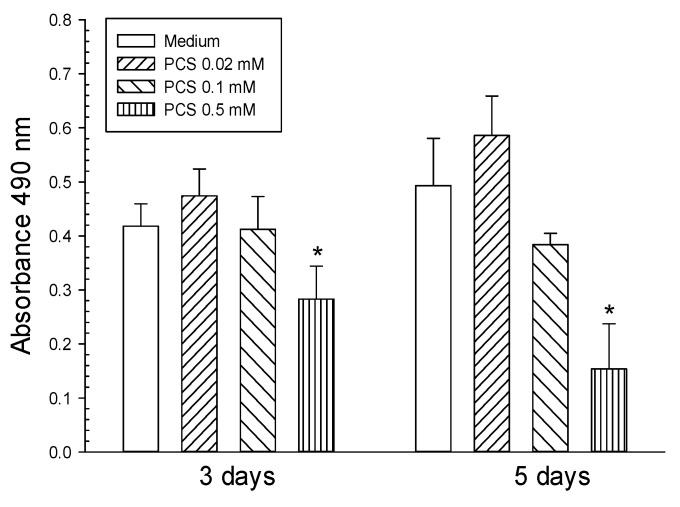
Cytotoxic effects of p-cresylsulfate (PCS) on human umbilical vein endothelial cells (HUVECs). HUVECs were treated with 0.02, 0.1, or 0.5 mM PCS for 3–5 days. The control group was treated with medium only. Cell viability (mean ± SD; n = 4 per group) was determined by the MTS assay. *Significant difference (*p* < 0.05) as compared with the controls.

To conduct studies on endothelial dysfunction induced by PCS exposure, the PCS compound was synthesized and its identity was also confirmed by electrospray ionization mass spectrometry (ESI-MS) as described previously [26]. The effects of various PCS doses on cell viability were determined using the MTS assay. A high dose (0.5 mM) of PCS treatment for 3–5 days reduced proliferation of ECs (Figure 1). In contrast, lower PCS doses (0.02 and 0.1 mM) had no effects on cell survival.

**Figure 2:**
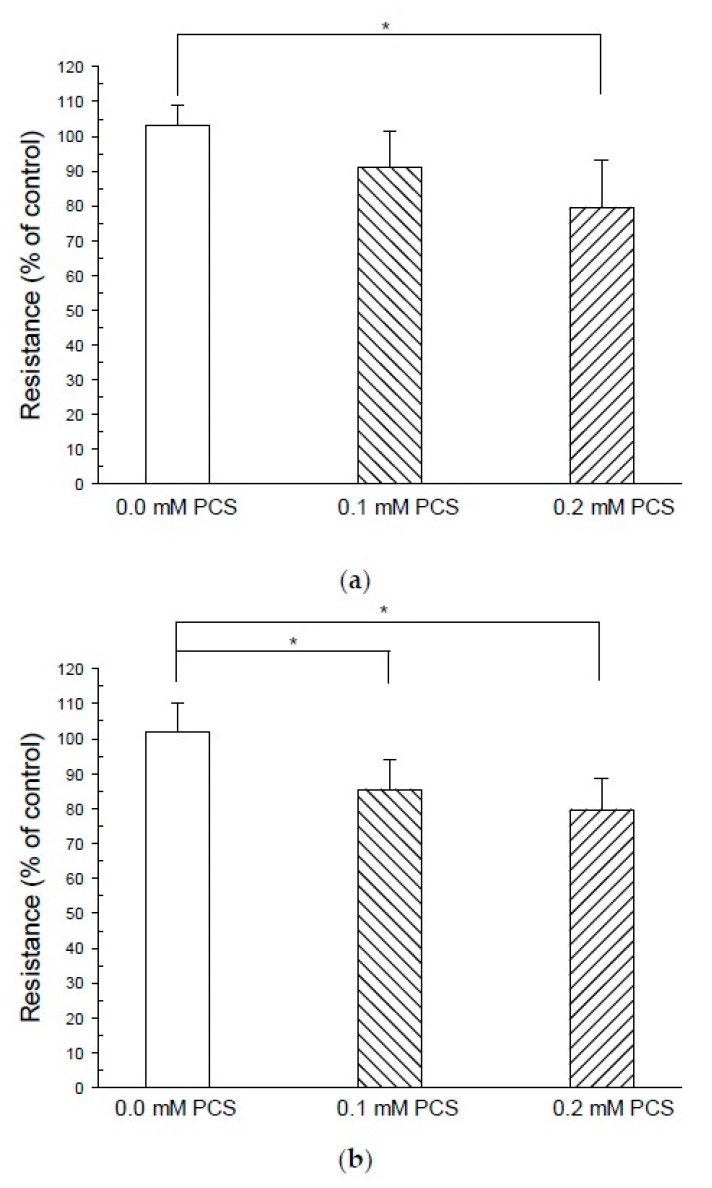
Treatment with p-cresylsulfate (PCS) decreases transendothelial electrical resistance (TEER). Human umbilical vein endothelial cells were grown on fibronectin-coated Transwell insert membranes (0.4 μm pore) for 48 h and treated with PCS for 48 or 72 h. The control group was treated with medium only. TEER was measured using a Millicell ERS-2 voltohmmeter (Millipore, Burlington, MA, USA) before PCS treatment and at the end of the experiment at 2 or 3 days. Resistance values [mean ± SD; n = 8 per group in the 48-h experiment (A); n = 10 per group in the 72-h experiment (B)] were normalized as the ratio of measured resistance to mean controls before receiving PCS treatment. *Significant difference (*p <* 0.05) as compared with controls.

To investigate whether PCS exposure led to alterations in endothelial permeability, we measured the changes in the transendothelial electrical resistance (TEER) of endothelial cell monolayers. The electrical resistance in growth medium-treated controls after 48 h displayed similar basal levels to the beginning of experiment. The PCS (0.1 mM) treatment for 48 h did not significantly decrease the electrical resistance as compared to the baseline of the control group (Figure 2). However, the treatment of 0.2 mM PCS decreased the electrical resistance over 48 h, resulting in a 14% reduction compared with the control group. Furthermore, both doses of PCS treatment decreased electrical resistance over a 72 h period, resulting in a 16% and 22% reduction, respectively, compared with the controls (Figure 2).

**Figure 3:**
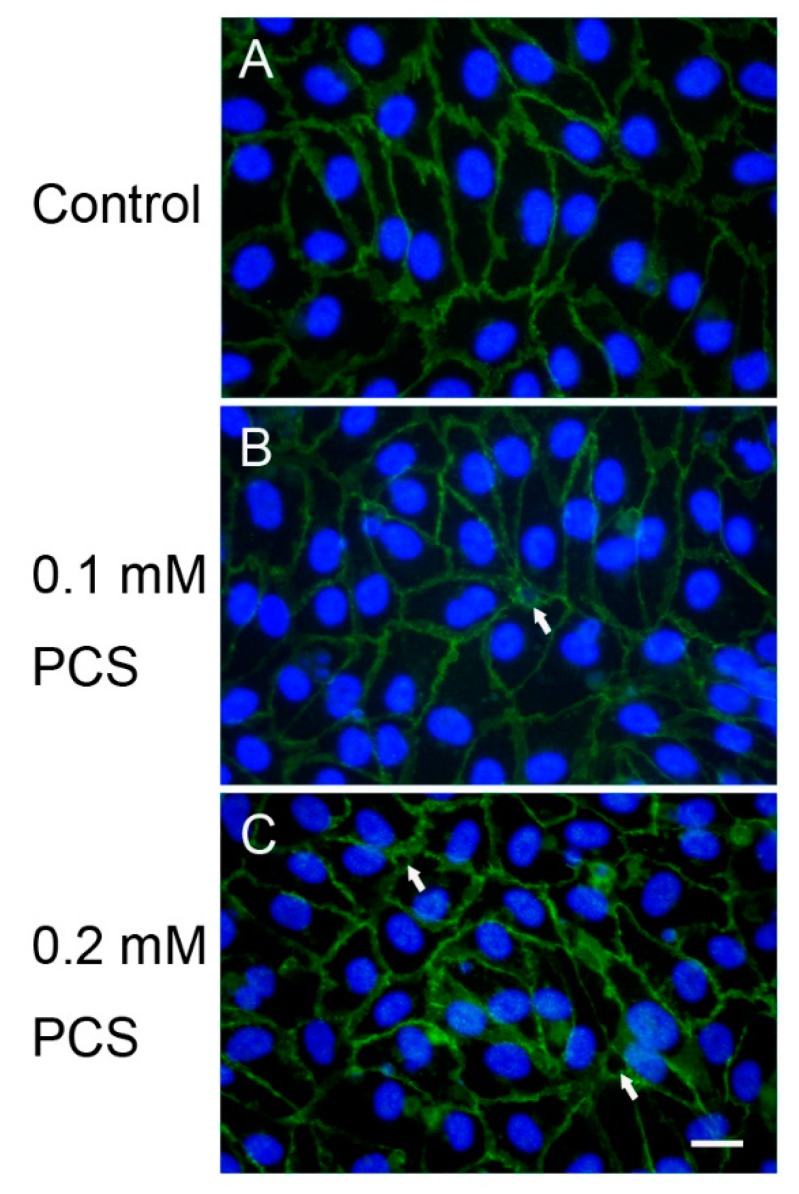
The effects of p-cresylsulfate (PCS) treatment on vascular endothelial (VE)-cadherin junctions and interendothelial gaps. Confluent human umbilical vein endothelial cell monolayers were treated with medium (control; A), 0.1 mM PCS (B), or 0.2 mM PCS (C) for 2 days. PCS treatment induced interendothelial gaps (indicated by arrows) visualized by immunofluorescence staining for VE-cadherin (green). The nuclei were stained with 4′,6-diamidino-2-phenylindole (blue). The images are representative of three independent experiments. Scale bar: 20 µm.

Interaction and binding of VE-cadherins on adjacent cells are essential for the formation of AJ architecture, and interendothelial gaps appear following the disruption of VE-cadherin adhesion. Alterations in VE-cadherin junctions and interendothelial gap formation of confluent HUVEC monolayers exposed to various doses of PCS for 48 h were detected by immunofluorescence staining for VE-cadherin. VE-cadherin staining in the untreated control cells for two days showed the integrity of the endothelial junctions (Figure 3). After 48 h of PCS treatment, interendothelial gaps with disruptions in cell-cell junctions were observed after treatment with 0.1 and 0.2 mM PCS (Figure 3).

**Figure 4:**
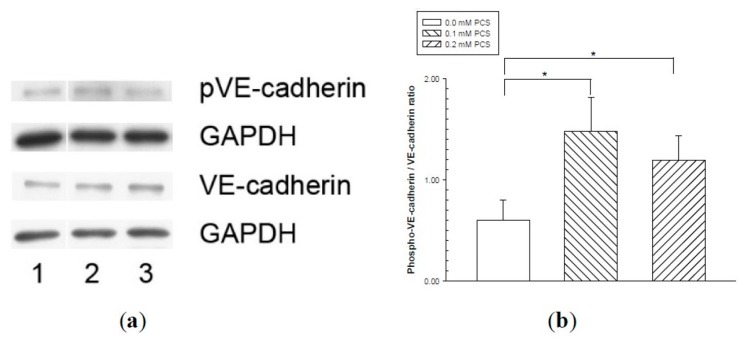
Vascular endothelial (VE)-cadherin tyrosine phosphorylation in endothelial monolayers treated with p-cresylsulfate (PCS). Human umbilical vein endothelial cell monolayers were treated with 0.1 or 0.2 mM PCS for 2 days. The control group was treated with medium only. Equal amounts (50 µg) of protein samples were loaded in duplicate gels and separated by SDS-PAGE. The protein levels of phosphorylated-VE-cadherin (pVE-cadherin), total VE-cadherin (VE-cadherin), and glyceraldehyde 3-phosphate dehydrogenase (GAPDH) were detected by western blotting. Following electrophoresis, the proteins were transferred to polyvinylidene fluoride membranes and detected by either anti-pVE-cadherin or anti-VE-cadherin antibodies via immunostaining on separate membranes. The levels of GAPDH in each membrane were also detected by anti-GAPDH antibodies. (a) Representative results. Lane 1: control sample; lanes 2–3: samples treated with PCS; lane 2: 0.1 mM PCS; and lane 3: 0.2 mM PCS. (b) Protein levels (mean ± SD, n = 3 per group) of pVE relative to VE.

**Figure 5:**
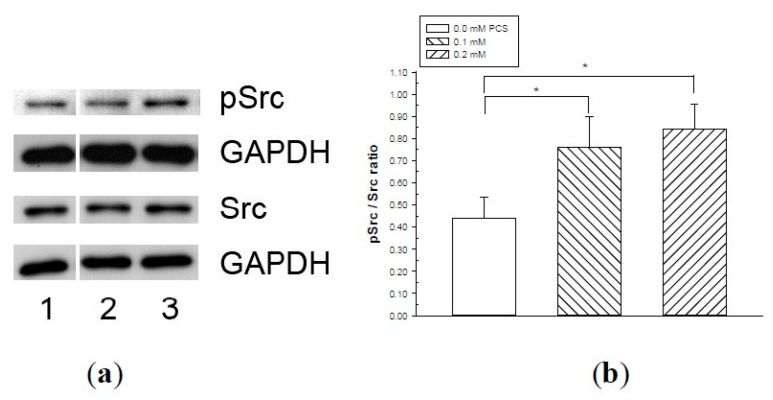
Src phosphorylation in endothelial monolayers treated with p-cresylsulfate (PCS). Human umbilical vein endothelial cell monolayers were treated with 0.1, or 0.2 mM PCS for 2 days. The control group was treated with medium only. Equal amounts (50 µg) of protein samples were loaded in duplicate gels and separated by SDS-PAGE. The protein levels of phosphorylated-Src (pSrc), total Src (Src), and glyceraldehyde 3-phosphate dehydrogenase (GAPDH) were detected by western blotting. Following electrophoresis, proteins were transferred to polyvinylidene fluoride membranes and detected by either anti-pSrc or anti-Src antibodies via immunostaining on separate membranes. The levels of GAPDH in each membrane were also detected by anti-GAPDH antibodies. (a) Representative results. Lane 1: control sample; lanes 2–3: samples treated with PCS; lane 2: 0.1 mM PCS; and lane 3: 0.2 mM PCS. (b) Protein levels (mean ± SD, n = 3 per group) of pSrc relative to Src.

Phosphorylation of VE-cadherin has been linked to increased endothelial permeability and disruption of intercellular junctions. We therefore determined whether PCS stimulation induced the phosphorylation of VE-cadherin at tyrosine 658 (Y658) based on previous studies [20,21] using western blotting. The amount of VE-cadherin was similar in all experimental groups (Figure 4). Increased levels of phosphorylated VE-cadherin were detected in HUVECs receiving various doses of PCS treatment compared with the controls (Figure 4). Src, one of the kinases, plays a role in the tyrosine phosphorylation of VE-cadherin [18,22,25]. Src activation is based on phosphorylation at tyrosine 416 (Y416), which is required to display its tyrosine kinase activity [27,28,29]; therefore, we used western blotting to determine whether the phosphorylated Src levels were increased by PCS stimulation. Figure 5 shows that the activated (phosphorylated) Src protein contents were significantly higher when the cells were treated with 0.1 and 0.2 mM PCS compared to the control group.

**Figure 6:**
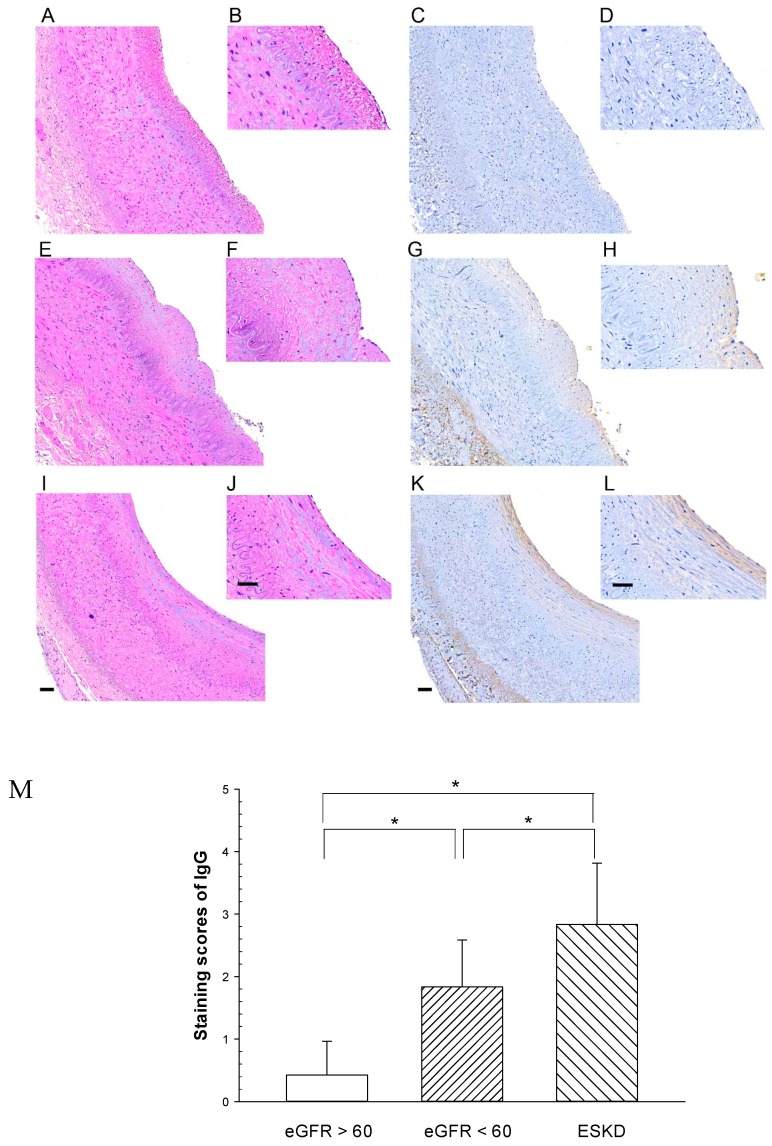
Representative immunohistochemical staining of IgG in CKD patient’s renal artery. Immunostaining for IgG was performed on transverse sections of three groups: (Figs. C, D) eGFR >60, (Figs. G, H) eGFR < 60 but not starting dialysis therapy, (Figs. K, L) end stage kidney disease (ESKD) on regular dialysis. The presence of IgG was indicated by brown reaction products with anti-IgG antibodies. Figs A, B, E, F, I, J: Hematoxylin/eosin staining in the adjacent sections. Large figures were displayed with a 20 × objective of the virtual microscopy software. Small figures were 2 times higher magnifications. Calibration bar = 50 um. Figure M IHC staining sores for IgG were shown on the *y*–axis (mean + SD; n = 6–7/group). * significant difference (*p* < 0.05).

To further elucidate the endothelial barrier integrity of the renal artery in CKD patients (Table 1), we next investigated whether endothelial leakage of IgG increased in the arterial intima. Patients were classified into three groups—group 1, eGFR > 60 (stage 1–2); group 2, eGFR < 60 but non starting dialysis therapy (stage 3–5); and group 3, end stage kidney disease (ESKD) on regular dialysis therapy (stage 5D). The patients’ CKD stages were also shown for each group. Significant differences of eGFR were observed among the three groups. We then investigated whether endothelial leakage of IgG increased in the arterial intima. The deposition of IgG was detected by brown staining products in the intima and periadventitia of the arterial wall by IHC. The objective of the study was whether arterial endothelial barrier dysfunction was induced by uremic toxins in CKD patients, therefore, we mainly focused on the microscopic evaluation of the deposition of IgG in the arterial intima and IgG deposition in the periadventitia was not included in the present study. Intense brown staining products of IgG deposition were detected in the intima including the endothelium and the sub-endothelial layer, indicating endothelial leakage of IgG (Figure 6). The immunostaining scores of the three groups of CKD patients are also shown in Figure 6. The results showed that the staining intensity of IgG in the arterial intima increased along with the decrease of eGFR of CKD patients with declining kidney function. Notably, IgG deposition was significantly higher inESKD dialysis patients than in other CKD groups.

## 3. Discussion

Although previous studies have indicated that free PC is associated with CVD in hemodialysis patients [30], PC is the metabolic precursor of PCS, and the majority of this compound in the blood is PCS. PCS was therefore synthesized to investigate endothelial dysfunctions related to PCS exposure. Previous animal studies have indicated that increased vascular permeability induced by PCS exposure is dose- and duration-dependent [26]. To access the effects of PCS on endothelial permeability, we measured the changes in the TEER of an endothelial cell monolayer exposed to PCS for 48 h or 72 h. Our results showed that a significant reduction in electrical resistance was detected following 0.2 mM PCS treatment over a 48 h period, but not at low-dose (0.1 mM) treatment. However, both doses of PCS decreased the electrical resistance over a 72-h period, indicating an increase in endothelial permeability with exposure to PCS. Interendothelial gaps were also observed in endothelial monolayers incubated with 0.1–0.2 mM PCS, but not in the controls. The PCS concentration (0.1–0.2 mM), containing 0.2–0.4 mg/L, used in the present study was similar to the serum PCS levels of CKD and ESKD dialysis patients [4,5,6]. Taken together, our results showed an increase in endothelial permeability accompanied by the disruption of interendothelial junctions, suggesting that 0.1–0.2 mM PCS treatment disrupted the endothelial monolayer.

Phosphorylation of VE-cadherin is involved in the disruption of inter-endothelial junctions [14,16,18,19,20,21]. In the present study, western blotting revealed an increase in phosphorylated VE-cadherin following treatment with 0.1 and 0.2 mM PCS, indicating that the disassociation of AJs with interendothelial gaps was related to the phosphorylation of VE-cadherin. Vasoactive agents, such as vascular endothelial growth factor (VEGF), tumor necrosis factor (TNF-α), thrombin, and histamine induce tyrosine phosphorylation of VE-cadherin [14,16,22,23,25]. Accordingly, tyrosine residues (eight to nine in humans and rodents) of the cytoplasmic terminus of VE-cadherin can be targets available for tyrosine kinases [13]. We then determined which tyrosines of VE-cadherin were phosphorylated during the induction of vascular permeability. It is known that the tyrosine residues Y645, Y658, Y685, Y731, and Y733 of VE-cadherin can be phosphorylated [13,18,20,31,32,33,34]. Recent studies have reported that Y685 phosphorylation of VE-cadherin is associated with vascular permeability, whereas Y731 phosphorylation is related to leukocyte extravasation [18,33,34,35]. Phosphorylation at Y658 of VE-cadherin is correlated with paracellular permeability, both in vivo and in vitro [20,32]. Our results also showed an increase in phosphorylated VE-cadherin (Y658) in endothelial cell monolayers after PCS treatment. Based on these results, future studies should explore the phosphorylation on other tyrosine residues of VE-cadherin and their related functions during PCS exposure.

The tyrosine phosphorylation of VE-cadherin is processed by various mechanisms including kinases [14,16,18,19,20,21]. Src, one of these kinases, plays a role in the tyrosine phosphorylation of VE-cadherin [18,22,25]. In endothelial cells, Src is localized with VE-cadherin in molecular complexes at AJs [24]. Recent studies have reported that Src is involved in barrier disruption in ECs challenged by inflammatory mediators and growth factors [14,16,22,23,25], and Src activation also results in tyrosine phosphorylation of VE-cadherin [18,22,24,25]. Our western blotting results showed that activated (phosphorylated) Src protein levels were significantly increased in PCS-exposed groups, indicating that activated Src was involved in the PCS-induced increase in endothelial permeability.

Previous studies demonstrated that PCS treatment increased the levels of endothelial microparticles in HUVECs [9]. Moreover, PCS has been shown to inhibit endothelial proliferation and wound repair in HUVECs [36]. Several reports have indicated that PCS induces the generation of reactive oxygen species (ROS) in leukocytes, proximal tubular cells, and endothelial cells [37,38,39]. PCS treatment also induces gap junction disruption in cultured cardiomyocytes [40]. In addition to PCS, PC also increases endothelial permeability associated with alterations in AJs, as demonstrated by the decreased staining of VE-cadherin [41]. Furthermore, IS, another protein-bound uremic toxin, has been shown to disrupt AJs in endothelial cells accompanied by the formation of intercellular gaps [42]. Our animal studies showed that PCS treatment increased vascular permeability [26]. In the present study, we showed that PCS induced the disruption of interendothelial junctions via Src-mediated phosphorylation of VE-cadherin. Taken together, these studies suggest that the protein-bound uremic toxins, PCS and IS, have multiple adverse effects on endothelial cells and the lining of the cardiovascular system, including heart chambers.

**Figure 7:**
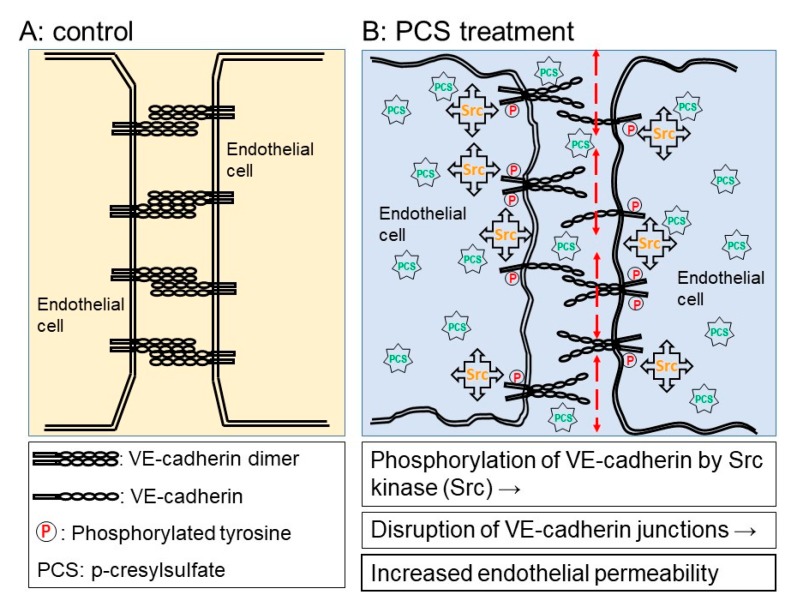
Schematic diagram of increased endothelial permeability in human umbilical vein endothelial cell monolayers exposed to p-cresylsulfate (PCS). (A) Control endothelial cells (ECs) exhibit endothelial barrier integrity with intercellular junctions. (B) In ECs exposed to PCS, disruption of the endothelial barrier following dissociation of endothelial adherens junctions is via Src-mediated phosphorylation of VE-cadherin.

In this study, we showed that PCS induced a significant increase in the endothelial permeability of the HUVEC monolayer, which was consistent with previous studies showing increased vascular permeability in microvessels when treated with PCS [26]. Moreover, the present study showed increased endothelial permeability associated with increased levels of phosphorylated VE-cadherin and phosphorylated Src in HUVECs exposed to PCS (Figure 7). However, a limitation of the present studies involved the use of endothelial cells derived from the human umbilical vein. Even though endothelial cell monolayers constitute the lining of blood vessels, endothelial cells display structural and functional heterogeneity along the blood vessels from microvessels to macrovessels, including the aorta [43,44,45]. Thus, further investigations are required to identify similar molecular mechanisms underlying increased endothelial permeability of endothelial cells isolated from human macrovessels such as the aorta or coronary artery. The results of IHC showed that advanced stages of CKD patients had significant increases in IgG deposition of in the intima including the endothelium and the sub-endothelial layer. The results of the IHC also showed that brown staining appeared in the peri-adventitia. It is likely that the IgG deposition in the peri-adventitia is due to vasa vasorum. Further studies may be warranted to investigate the IgG deposition in the peri-adventitia.

The mechanisms underlying Src activation by exposure to PCS remain elusive. Previous studies indicate that CKD patients are characterized by enhanced oxidative stress, appearing in the early stages and increasing with later stages, even in ESKD dialysis patients [5,46,47]. Indeed, PCS induces the generation of ROS, resulting in oxidative stress [37,38,39]. It is also known that oxidative stress can activate Src kinase [48,49]. Thus, it is likely that uremic toxins, including PCS, induce endothelial barrier dysfunction through Src activation by oxidative stress.

Increased vascular permeability is considered as a pathological indication of early vascular injury [50]. Previous studies indicate that there is an increased endothelial leakage of IgG in the arterial intima in the CKD rats with high serum levels of PCS [26]. Our data also indicate that the endothelial leakage of IgG in the arterial intima increased along with the declining kidney function of CKD patients. A further increase in IgG deposition was noted in ESKD dialysis patients. Although we did not measure serum PCS levels in CKD patients, it is known that serum PCS levels are significantly higher in CKD patients even in ESKD patients with dialysis treatment compared with healthy controls [4,5,6]. Taken together, these studies indicate that the endothelial barrier dysfunction with increased endothelial leakage in CKD patients is induced by uremic toxins, including PCS.

CVD is the main cause of morbidity and mortality in CKD patients worldwide [51]. The retention of renally excreted molecules called uremic toxins is involved in the pathogenesis of CVD [5,10,52]. Increased endothelial permeability related to endothelial barrier dysfunction plays a role in the development of CVD, including atherosclerosis [53]. Our previous studies showed increased endothelial leakage induced by PCS in skin microvessels and the aorta of CKD rats [26]. Moreover, our results also showed that increased levels of PCS induced disruption of interendothelial junctions accompanied by the increased endothelial permeability and increased endothelial leakage of IgG in the renal arteries in CKD and ESKD dialysis patients. Increased endothelial permeability may therefore contribute to the increased cardiovascular disease risk associated with CKD. It is known that protein-bound uremic toxins cannot be efficiently removed by currently available hemodialysis [1,2,10]. Alternative techniques for the efficient removal of uremic toxins, including PCS and IS, from protein-bound molecules may be possible as therapeutic strategies to prevent increased endothelial permeability associated with endothelial barrier dysfunction in CKD and ESKD dialysis patients.

## 4. Materials and Methods

### 4.1. Synthesis of P-Cresylsulfate (PCS)

The sodium salt of PCS was synthesized as described [26]. The purity and identity of synthetic PCS were confirmed by high-performance liquid chromatography analysis and ESI-MS as previously described [26].

### 4.2. Cell Culture and Cell Viability after PCS Treatment

HUVECs from Lonza (Walkersville, MD, USA) were cultured in endothelial basal medium-2 containing the Endothelial Cell Growth Medium Supplement BulletKit (Lonza) in a 5% CO_2_ incubator at 37 °C. The cells were passaged with trypsin-EDTA, trypsin neutralizing solution, and HEPES buffer solution (Lonza) every 3–4 days. The experiments were performed between passages 4 and 6.

For cell viability, HUVECs were seeded at 1.5 × 10^4^ cells/well in 96-well plates overnight prior to the experiment. HUVECs were treated with PCS (0, 0.02, 0.1, or 0.5 mM) for 3–5 days. The medium with or without PCS was changed every two days. The effects of PCS on the viability of HUVECs were measured using the MTS assay (Promega, Madison, WI, USA). After PCS exposure, the cells were incubated with fresh medium (phenol red-free) containing MTS reagent for 4 h, followed by absorbance measurements at 490 nm.

### 4.3. TEER

The endothelial barrier properties were accessed with TEER measurements using an electrical resistance system (Millicell ERS-2; Merck Millipore, Burlington, MA, USA). To optimize cell adhesion during the experiments, the Transwell inserts (6.5 mm with 0.4 µm pores; Corning, Corning, NY, USA) were coated with fibronectin. Fibronectin (BD Biosciences, San Jose, CA, USA) was dissolved in MilliQ water as a stock solution to a concentration of 1 mg/mL. The fibronectin stock solution was diluted, added to Transwell inserts at a density of 7.5 µg/cm^2^, and incubated in a 5% CO_2_ incubator at 37 °C for 2 h. The solution was then removed, and Transwell inserts were dried for 30 min in a laminar flow hood. HUVEC growth medium was added to the Transwell inserts, and the wells were incubated for 30 min at 37 °C prior to cell seeding. The HUVECs were seeded at 5 × 10^4^ cells/cm^2^ in the HUVEC growth medium in Transwell inserts (6.5 mm with 0.4 µm pores) and grown to confluence for 24–48 h prior to the experiments. The HUVECs inside Transwell inserts were treated with 0.1 mM or 0.2 mM PCS for 2 or 3 days in a 5% CO_2_ incubator at 37 °C. The control group was treated with medium (without PCS) only. TEER was measured using a Millicell ERS-2 Voltohmmeter (Merck Millipore) according to the manufacturer’s protocol before PCS treatment and at the end of the experiment at 2 or 3 days. Blank TEER values were also measured in Transwell inserts without cells and were subtracted from the measured values of samples. The resistance values (Ω cm^2^) were then normalized as the ratio of measured resistance to mean controls before PCS treatment.

### 4.4. Immunofluorescence Microscopy

Cell culture chamber slides (eight wells; SPL Life Sciences, Porcheon-si, Korea) were coated with fibronectin (BD Biosciences, San Jose, CA, USA) at a density of 1.5 µg/cm^2^ and incubated for 1 h at 37 °C. The solution was then removed and the chamber slides were dried for 1 h in a laminar flow hood. The HUVECs were seeded at 5 × 10^4^ cells/cm^2^ in a HUVEC growth medium in cell culture slides and were grown to confluence for 24–48 h prior to the experiment. The HUVECs were treated with 0.1 mM or 0.2 mM PCS for 2 days in a 5% CO_2_ incubator at 37 °C. The control group was treated with the medium only. At the end of the experiments, the cells were washed three times with phosphate-buffered saline (PBS) and fixed with 4% paraformaldehyde in PBS for 20 min at room temperature (RT). After washing three times with PBS, the cells were permeabilized for 1 h at RT with PBS containing 0.3% Triton X-100 and 5% fetal bovine serum. The cells were then washed three times with PBS and incubated for 2 h at RT with rabbit anti-VE cadherin polyclonal antibodies (1:100; sc-28644; Santa Cruz Biotechnology, Fort Worth, TX, USA) in PBS containing 0.3% Triton X-100 and 1% bovine serum albumin, followed by Alexa Fluor 488-labeled secondary antibodies (1:100; 111-545-003; Jackson ImmunoResearch Laboratories, West Grove, PA, USA) for 1 h at RT. After washing three times with PBS, the nuclei were counterstained using 4′,6-diamidino-2-phenylindole (Molecular Probes, Eugene, OR, USA) for 5 min at RT according to the manufacturer’s protocol. The chambers were removed and the slides were covered with Vectashield mounting medium (Vector Laboratories, Burlingame, CA, USA) and coverslips. Fluorescence images were acquired using a Nikon E600 fluorescence microscope with a Nikon DS-Fi1c digital color camera (Nikon, Tokyo, Japan).

### 4.5. Western Blotting

Confluent monolayers of HUVECs were treated with either the control or 0.1 mM or 0.2 mM PCS for 2 days. HUVECs treated with PCS were lysed using a cell lysis solution containing complete protease inhibitors, processed for protein extraction, and the protein content was quantitated using the Lowry method as previously described [54]. The protein expression was quantitated by western blotting as previously described [54]. Briefly, the proteins were separated by SDS-PAGE. Following electrophoresis, the proteins were transferred to polyvinylidene fluoride membranes (Hybond-P; GE Healthcare, Singapore). The membranes were soaked in blocking solution containing 5% skim milk and incubated overnight at 4 °C with antibodies specific to the target proteins. The target proteins were the following—VE-cadherin (rabbit polyclonal antibody, sc-28644, Santa Cruz Biotechnology); phosphor-VE-cadherin-Tyr658 (rabbit polyclonal antibody, 44-1144G, Invitrogen, Carlsbad, CA, USA); phosphor-Src-Tyr416 (rabbit polyclonal antibody, 2101, Cell Signaling Technology, Danvers, MA, USA); Src (rabbit polyclonal antibody, 2108, Cell Signaling Technology); and glyceraldehyde 3-phosphate dehydrogenase (GAPDH; rabbit polyclonal antibody, ABS 16; Millipore) as an internal control. The membranes were then incubated with horseradish peroxidase-conjugated secondary antibodies (AP 132p; Millipore), visualized using enhanced chemiluminescence, and analyzed by densitometry. The levels of GAPDH in each sample were detected using anti-GAPDH polyclonal antibodies. The levels of target proteins relative to GAPDH were used to calculate the ratio between the phosphorylated form (phosphorylated-VE-cadherin, pVE-cadherin; phosphorylated-Src, pSrc) and the total form (VE-cadherin; Src) in the PCS and control groups.

### 4.6. Microscopic Evaluation of the Endothelial Barrier of the Renal Artery of CKD Patients

The study participants were individuals with an estimated glomerular filtration rate (eGFR) 2.7–89.9 mL/min/1.73 m^2^ and consecutively treated in at the Department of Nephrology at the (Chi Mei) medical center. The study protocol (IRB, 10612-015) was approved by the institutional review of Chi Mei Medical Center. Informed consent was obtained from all the subjects. The patients were also classified into three groups—eGFR > 60 mL/min/1.73 m^2^ (stage 1,2); eGFR< 60 mL/min/1.73 m^2^ but non starting dialysis therapy (stage 3–5); end stage kidney disease (ESKD) on regular dialysis therapy (stage 5D). These renal artery samples were from patients who received unilateral total nephrectomy for malignant tumors.

After nephrectomy, the renal artery segments associated with the diseased kidney were immediately fixed in 10% formalin. The formalin–fixed specimens were processed for paraffin embedding and 3 µm sections were prepared. The procedure of immunohistochemical staining was designed as according to the manufacturer’s protocol (TAlink mouse/rabbit polymer detection system; TAHC04D, BioTnA Biotech, Kaohsiung, Taiwan). In brief, deparaffinized and hydrated tissue sections were incubated in a hydrogen peroxidase block (BioTnA, Kaohsiung, Taiwan), followed by antigen retrieval in a boiling citrate buffer (pH 6.0; BioTnA, Kaohsiung, Taiwan). The sections were then incubated with a blocking solution (BioTnA, Kaohsiung, Taiwan) for 30 min, and mouse monoclonal anti-Human IgG antibodies (1:1000; cat no.23001, Leadgene, Tainan, Taiwan) for one hour. The sections were then incubated with rabbit anti-mouse antibodies (TAlink post primary, BioTnA, Kaohsiung, Taiwan) and followed by HRP-conjugated polymer (TAlink polymer, BioTnA, Kaohsiung, Taiwan). Every staining step was followed by rinsing in PBS (0.1 M) for two min (three times). The sections were then incubated with a 3,3′-diaminobenzidine (DAB) solution (BioTnA, Kaohsiung, Taiwan) for one minute, rinsed, and counterstained with hematoxylin. The sections were then rinsed in water, dehydrated, cleared in xylene, and mounted with coverslips. Hematoxylin/eosin staining was also performed in the adjacent sections. The procedure of digital conversion of tissue slides to digital slides was performed as previous studies [26]. The renal artery segments were not collected due to the surgical procedure in some patients, therefore six samples were in groups 2–3 and seven samples were in group 1 for IgG IHC staining.

The objective of the study was whether arterial endothelial barrier integrity in CKD patients was intact, therefore, we mainly focused on the deposition of IgG in the arterial intima and IgG staining in the periadventitia was not included for comparison. The staining intensity of the arterial intima (AI) including endothelial cells was scored according to 6 grades—0 (no staining), 1 (1–5% of AI with staining), 2 (6–25% of AI with staining), 3 (26–50% of AI with staining), 4 (51–75% of AI with staining), and 5 (>75% of AI with staining). The staining of IgG IHC and hematoxylin/eosin and staining findings were implemented, scored, and reviewed by a pathologist at Litzung Biotechnology INC (Kaohsiung, Taiwan).

### 4.7. Statistical Analyses

The values are expressed as the mean ± SD. An analysis of variance (ANOVA) on ranks was used to compare the means of the different treatment groups. When the ANOVA using ranks test indicated an overall significant difference (*p* < 0.05), a Dunnett or Student-Newman-Keuls pairwise multiple comparison test was used to determine the statistical significance of the differences between any groups. The differences were considered statistically significant at *p* < 0.05.

## Figures and Tables

**Table 1:** Estimated glomerular filtration rate (eGFR) of CKD patients.

Variable	Group 1 (n = 7)	Group 2 (n = 7)	Group 3 (n = 7)	*p* Value
Age (years)	69.7 ± 12.6	76.7 ± 5.1	62.1 ± 11.4	0.011
eGFR, mL/min/1.73 m^2^	73.4 ± 10.5	33.9 ± 15.9	6.4 ± 2.5	*<0.001
